# Risk-adaptive paradigm for focal versus whole-gland salvage treatment for radio-recurrent prostate cancer

**DOI:** 10.3389/fonc.2022.998390

**Published:** 2022-09-29

**Authors:** Martin T. King, David D. Yang, Anthony V. D’Amico, Ivan Buzurovic, Thomas C. Harris, Christian V. Guthier, Graeme S. Steele, Martin N. Kathrins, Atish D. Choudhury

**Affiliations:** ^1^ Department of Radiation Oncology, Brigham and Women’s Hospital, Boston, MA, United States; ^2^ Department of Urology, Brigham and Women’s Hospital, Boston, MA, United States; ^3^ Department of Medical Oncology, Dana-Farber Cancer Institute, Boston, MA, United States

**Keywords:** radiorecurrent prostate cancer, SBRT, brachytherapy, MRI, PSMA

## Introduction

For patients undergoing dose-escalated external beam radiation therapy (EBRT) for the treatment of prostate cancer, National Comprehensive Cancer Network (NCCN) prostate cancer guidelines recommend short-term androgen deprivation therapy (ADT) (4-6 months) for unfavorable intermediate risk disease, and long-term ADT (18-36 months) for high risk disease (1). However, a sizeable portion of patients who receive such standard-of-care (SOC) treatment remain at risk for biochemical recurrence (BCR). In a recent update of the DART 01/05 trial, in which 355 patients receiving dose-escalated EBRT were randomized to short-term (4 months) versus long-term (28 months) ADT, the 10-year rate of biochemical progression-free survival (bPFS) for the intermediate risk cohort who received standard-of-care (SOC) short-term ADT was 73%. The corresponding rate for high risk cohort who received SOC long-term ADT was 67% (2).

Retrospective and prospective studies utilizing prostate-specific membrane antigen (PSMA) positron emission tomography (PET) have suggested that a sizeable portion of patients with BCR after RT have an intraprostatic recurrence. A large meta-analysis of post-RT BCR patients imaged with 68Ga-PSMA PET reported a local failure rate of 52% (3). An early prospective series of 130 patients with post-RT BCR and imaged with 18F-DCFPyL, reported a 62.9% rate of local failure (4). A follow-up study of 93 patients treated with brachytherapy (BT) +/- EBRT reported a similar local failure (prostate and/or seminal vesicles) rate of 62.8%, with an isolated local failure rate of 46.5%. Interestingly, isolated local failures were noted in 54.3% of monotherapy patients, compared to only 12.5% of combination (EBRT + BT) therapy patients (5). Another study of 79 patients with post-RT BCR reported an isolated local failure rate of 48%, as detected by 18F-DCFPyL (6).

The increased ability of PSMA PET to detect local recurrences may increase the demand for definitive local salvage treatments, especially for those with isolated disease. Local salvage therapy is an attractive option because it may be curative in some instances (7, 8). If not curative, definitive local therapies may delay the onset and lifetime exposure of salvage ADT, which has been associated with declines in quality-of-life (9) and increased cardiovascular risk, especially for those with pre-existing comorbidities (10).

## Whole-gland salvage therapy

However, there are a few important points that need to be made about local salvage therapy, particularly non-surgical approaches, which have been associated with less toxicity than salvage prostatectomy in a recent meta-analysis (7). We focus our discussion on Radiation Therapy Oncology Group (RTOG) 0526, given that this was a cooperative group, single-arm, phase 2 trial with a minimum 5-year follow-up (8). In this trial, 100 patients across 20 centers without systemic disease were treated with whole-gland low dose-rate (LDR) prostate brachytherapy. 16% of patients received ADT.

First, local whole-gland salvage therapy can be toxic for a small, but significant patient subset. In RTOG 0526, the rate of late grade 3 gastrointestinal/genitourinary adverse events (AEs) was 14%, which resided between the pre-specified acceptable (<=10%) and unacceptable (>=20%) thresholds. The only factor associated with grade 3+ toxicity was prostate V100 (fractional volume of the prostate that receives 100% of the prescription dose), suggesting that less-than-full gland therapy may be associated with a lower risk of clinically significant, late toxicity (11).

Second, the long-term biochemical control with local whole-gland salvage therapy is quite modest. The 10-year bPFS for RTOG 0526 was 46%, despite comprehensive prostate coverage (median prostate V100 94%, D90 (minimum dose received by 90% of the prostate) 109%), and conservative enrollment criteria (initial diagnosis of low- or intermediate-risk prostate cancer, post-EBRT PSA < 10 ng/dL, and negative systemic staging with bone and CT scans) (8). Whether improved results may be achieved with advanced imaging (multi-parametric magnetic resonance imaging (mpMRI), advanced PET (6)) for staging, or more systemic ADT utilization remain open questions. Nevertheless, the modest long-term oncologic control makes toxicity considerations more paramount.

Third, the majority of patients with BCR do not die from prostate cancer (9). In RTOG 0526, only 4 of 14 deaths were from known prostate cancer (10-year disease-free survival of 70%). As a result, quality-of-life becomes an important consideration in choosing a treatment strategy for recurrent disease. Some patients with local-only recurrences who are concerned of treatment-related side-effects, such as urinary toxicity with local salvage therapy or fatigue/hot flashes/cardiovascular events with salvage ADT, may reasonably opt for surveillance.

## Focal salvage therapy

Focal salvage therapy is an alternative to whole-gland salvage treatment. The premise of focal therapy is that local recurrences after RT often occur at the site of the initial primary tumor or dominant intraprostatic lesion (DIL) (12, 13), as noted on mpMRI. Treatment of the residual DIL at the time of recurrence would address the bulk of the disease, while sparing the rest of the prostate and adjacent organs-at-risk from treatment-related toxicities.

Focal therapy has been shown to be well-tolerated across all modalities. Low rates (<=10%) of significant grade 3+ GI/GU toxicities have been reported for focal LDR brachytherapy (14, 15), single-fraction (16–18) and two-fraction (19) HDR brachytherapy, and SBRT (20–22). Furthermore, studies involving high-intensity focused ultrasound (HIFU) (23) and cryotherapy (24) have reported reduced toxicity rates of focal compared with whole-gland treatment.

However, focal therapy may be associated with a greater risk of intraprostatic recurrence. This is because of persistent limitations with advanced imaging modalities. A recent study of 68Ga-PSMA PET reported 100% sensitivity for identifying the DIL prior to salvage prostatectomy. Yet, smaller lesions were missed in cases with multifocal relapse (25). Furthermore, MRI has been shown to miss small lesions and underestimate lesion size in the radiorecurrent setting (26). Although MRI and PSMA PET may provide complementary information regarding lesion location (27), further multi-modality target delineation studies, preferably against a pathologic gold-standard, are needed.

In addition, the risk of intra-prostatic, out-of-field, recurrence after focal therapy has not been well-characterized. In a series of 50 patients treated with single fraction, focal HDR brachytherapy, 6 patients experienced an intraprostatic, out-of-field recurrence, as detected on 68Ga-PSMA-PET (17). In another study of 30 patients, who received two fraction, focal HDR brachytherapy, 3 of 29 patients with a post-treatment MRI had radiographic, but not biopsy-proven, evidence of failure within or around the site of focal salvage (19). Though early oncologic results of focal therapy appear promising (14, 19), prospective series with standardized target delineation strategies based on contemporary imaging modalities and long-term follow-up are needed. If responses are durable, this may further increase enthusiasm for focal therapy.

## A risk-adaptive paradigm for whole-gland versus focal therapy

**Figure 1:**
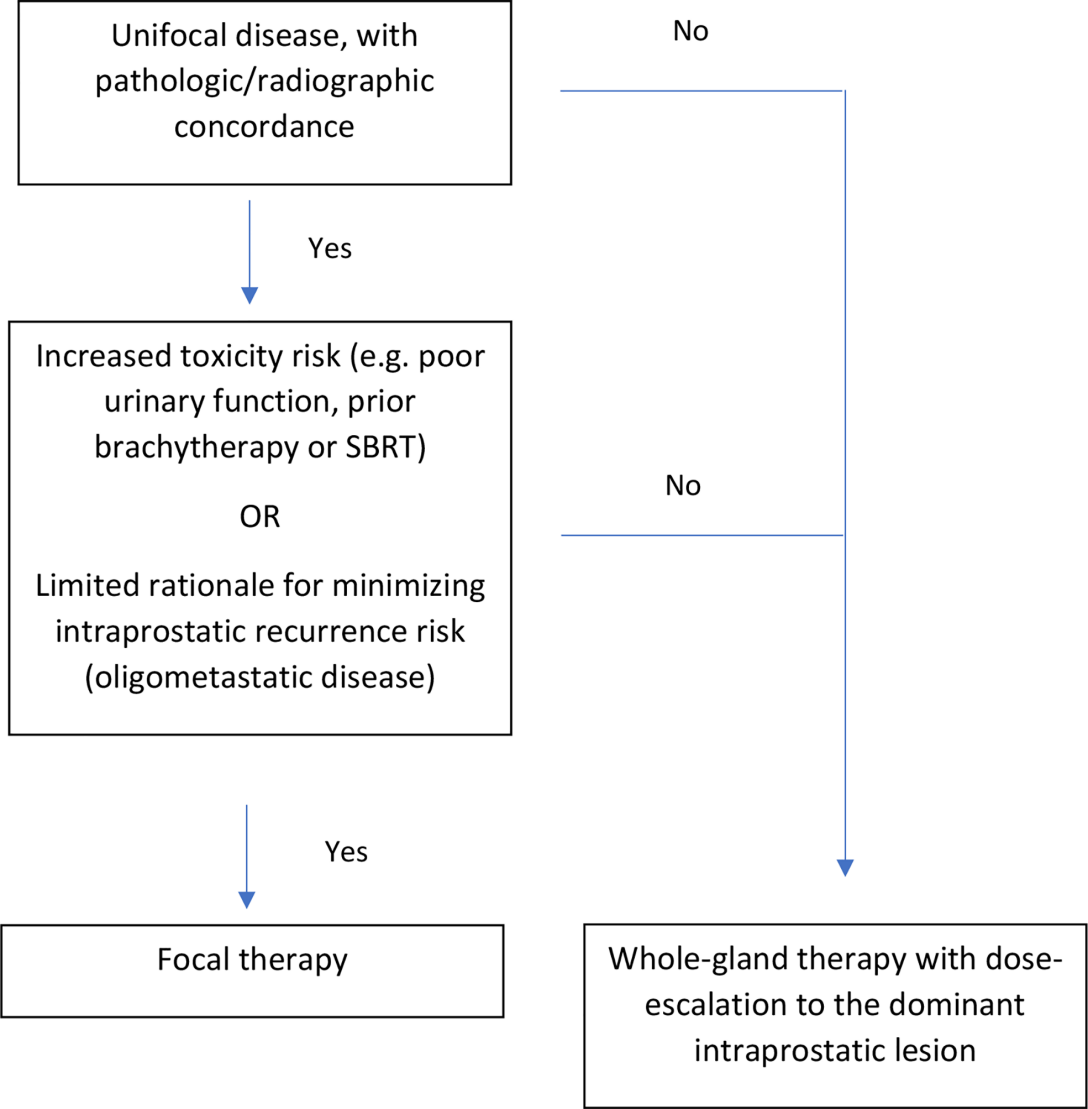
Risk-adaptive approach for focal versus whole-gland salvage therapy for radiorecurrent prostate cancer.

We believe that a risk-adaptive approach may balance the reduced risk of intraprostatic recurrence with whole-gland therapy against the reduced risk of toxicity with focal-therapy (see **Figure 1**). Prior to implementing the risk-adaptive approach, we would recommend that patients undergo a comprehensive assessment of their prior prostate cancer treatment, and current genitourinary/gastrointestinal function. Furthermore, the location of intraprostatic disease should be fully characterized with PSMA PET, mpMRI, as well as systematic plus targeted biopsies.

Ideal candidates for focal therapy should have unifocal disease, with pathological/radiographic concordance. In other words, biopsies corresponding to the DIL should show pathologic disease, whereas biopsies far from the DIL should not. Furthermore, candidates should exhibit either: 1) a high-risk of toxicity with whole-gland re-irradiation (e.g. prior history of grade 3 urinary toxicity, poor urodynamic testing (peak flow rate < 10 cc/s, post-void residual volume > 100 cc) (28, 29), prostate size > 40 cc (11), prior LDR/HDR brachytherapy or SBRT (30)) or 2) limited rationale for minimizing intraprostatic recurrence risk (e.g. oligometastatic disease). For oligometastatic disease, metastasis-directed therapy (MDT) (31) could be accomplished by treating the focal intraprostatic lesion plus the distant sites. There is limited rationale for treating the entire prostate, given that the risk of distant progression after MDT likely supersedes the risk of intraprostatic recurrence.

Patients who are not ideal candidates for focal therapy could elect to undergo whole-gland therapy with dose-escalation to the DIL. For both cases, we would recommend contouring the DIL on mpMRI (T2, diffusion-weighted, and contrast-enhanced) and prostate PET. The contoured DIL, which should encompass all areas of suspicious disease across imaging modalities, can then be fused to the imaging modality utilized for treatment (e.g. transrectal ultrasound for LDR/HDR brachytherapy, or MRI for SBRT). The treatment should then be delivered according to standard practices, with careful attention to organ-at-risk metrics.

We currently have an activated phase 2 protocol testing this risk-adaptive approach for radiorecurrent prostate cancer utilizing salvage two-fraction HDR brachytherapy. This protocol, which falls under a master protocol of MRI simulation (NCT 04545957), will evaluate whether the proportion of patients with an EPIC-26 urinary decline exceeding twice the minimally important difference (12 points) is less than 30% at 2-years. We plan to accrue 46 patients for this protocol.

In this protocol, patients will receive 10.5 Gy x 2 fractions to the DIL gross tumor volume (GTV) + 5 mm margin if eligible for focal therapy according to the criteria delineated above. Otherwise, patients will receive 10.5 Gy x 2 fractions to the entire gland. In both cases, patients will have dose-escalation to the DIL clinical target volume (CTV), defined as the DIL GTV + 3 mm, to >115% (~12.1 Gy) of the prescription dose. The utilization of ADT is given per physician discretion. However, our clinical practice tends to include 6-months of ADT, especially if PSA doubling time <= 1 years, due to the oncologic benefit noted when ADT is given with post-prostatectomy RT (32, 33).

In summary, a risk-adaptive approach for salvage re-irradiation may allow for patients at higher risk of urinary toxicity to better preserve their urinary quality-of-life with focal therapy. Such patients can still receive definitive treatment to the gross disease, but may carry a greater risk of intraprostatic recurrence. Further prospective studies are necessary to improve this risk stratification approach and define the ideal candidates for focal salvage therapy.

## Author contributions

MK drafted the manuscript. DY, AD, IB, TH, CG, GS, MK, and AC reviewed the manuscript. All authors contributed to the article and approved the submitted version.

## Conflict of interest

The authors declare that the research was conducted in the absence of any commercial or financial relationships that could be construed as a potential conflict of interest.

## Publisher’s note

All claims expressed in this article are solely those of the authors and do not necessarily represent those of their affiliated organizations, or those of the publisher, the editors and the reviewers. Any product that may be evaluated in this article, or claim that may be made by its manufacturer, is not guaranteed or endorsed by the publisher.
